# Pediatric Inflammatory Multisystem Syndrome: Statement by the Pediatric Section of the European Society for Emergency Medicine and European Academy of Pediatrics

**DOI:** 10.3389/fped.2020.00490

**Published:** 2020-08-28

**Authors:** Ruud G. Nijman, Ann De Guchtenaere, Berthold Koletzko, Rob Ross Russell, Sian Copley, Luigi Titomanlio, Stefano del Torso, Adamos Hadjipanayis

**Affiliations:** ^1^Section of Paediatric Infectious Diseases, Department of Infectious Diseases, Faculty of Medicine, Imperial College London, London, United Kingdom; ^2^Department of Paediatrics, Ghent University, Ghent, Belgium; ^3^Dr. von Hauner Children's Hospital, Ludwig-Maximilians-Universität München, Munich, Germany; ^4^Cambridge University Hospitals NHS Foundation Trust, Cambridge, United Kingdom; ^5^Health Education North East, Newcastle Upon Tyne, United Kingdom; ^6^Paediatric Emergency Department, Hopital Universitaire Robert-Debre, Paris, France; ^7^Studio Pediatrico, Padova, Italy; ^8^Department of Medicine, European University Cyprus, Nicosia, Cyprus

**Keywords:** children, COVID-19, SARS-CoV-2, PIMS-TS, MIS-C, Kawasaki-like disease, fever

## Abstract

A rise in cases with a new hyperinflammatory disease in children has been reported in Europe and in the Unites States of America, named the Pediatric Inflammatory Multisystem Syndrome—temporally associated with SARS-CoV-2 (PIMS-TS). There appears to be a wide spectrum of signs and symptoms with varying degrees of severity, including a toxic shock like presentation with hypovolaemia and shock, and a Kawasaki-like presentation with involvement of the coronary arteries. Most of these children have evidence of a previous infection with SARS-CoV-2, or a history of significant exposure, but not all. Limited data exist on the incidence of PIMS-TS, but it remains a rare condition. Early recognition and escalation of care is important to prevent the development of serious sequelae, such as coronary artery aneurysms. Clinicians assessing febrile children in primary and secondary care should include PIMS-TS in their differential diagnoses. In children fulfilling the case definition, additional investigations should be undertaken to look for evidence of inflammation and multiorgan involvement. Suspected cases should be discussed with experts in pediatric infectious diseases at an early stage, and advice should be sought from critical care in more severe cases early. There is limited consensus on treatment; but most children have been treated with immunoglobulins or steroids, and with early consideration of biologicals such anti-TNF and anti-IL1 agents. Treatment should ideally be within the context of controlled treatment trials. Clinicians are encouraged to document and share their cases using research registries.

## Statement

According to the currently available evidence, SARS-CoV-2 infection in children is rarely associated with severe disease (1), children are less likely to be infected compared with adults, and children are likely to be less infectious compared with infective adults (2, 3). However, multiple cases of children with a new hyperinflammatory condition in children subsequent to SARS-CoV-2 infection have been reported in Europe and the United States of America (Tables 1, 2).

**Table 1:** Overview of studies reporting children with PIMS-TS.

	**Population**	**Location**	**Number of children in study**	**Number of settings**	**Eligibility**	**Date of publication**
Riphagen et al. (4)	Children with hyperinflammatory shock	London, UK	8	1	Admitted to PICU	May 6th 2020
Verdoni et al. (5)	Children with Kawasaki-like disease	Bergamo, Italy	10	1	Admitted to the general pediatric unit	May 13th 2020
Belhadjer et al. (6)	Children with acute heart failure in MIS-C	France, Switzerland	35	14	Admitted to PICU	May 17th 2020
Chiotos et al. (7)	Children with MIS-C	Philadelphia, US	6	1	Admitted to PICU	May 28th 2020
Grimaud et al. (8)	Acute myocarditis and multisystem inflammatory emerging disease in critically ill children	Paris, France	20	4	Admitted to PICU	June 1st 2020
Toubiana et al. (9)	Children with Kawasaki-like multisystem inflammatory syndrome	Paris, France	21	1	Admitted to general pediatric ward	June 3rd 2020
Miller et al. (10)	Children with MIS-C	New York, US	44	1	Admitted to hospital	June 4th 2020
Whittaker et al. (11)	Children with PIMS-TS	United Kingdom	58	8	Admitted to hospital	June 8th 2020
Cheung et al. (12)	Children with MIS-C	New York, US	17	1	Admitted to hospital	June 8th 2020
Capone et al. (13)	Children with MIS-C	New York, US	33	1	Admitted to hospital	June 10th 2020
Pouletty et al. (14)	Children with PIMS-TS mimicking Kawasaki disease (Kawa-COVID-19)	Paris, France	16	7	Admitted to hospital	June 11th 2020
Ramcharan et al. (15)	Children with PIMS-TS	Birmingham, UK	15	1	Admitted to hospital	June 12th 2020
Kaushik et al. (16)	Children with MIS-C	New York, US	33	3	Admitted to PICU	June 14th 2020
Riollano-Cruz et al. (17)	Children with MIS-C	New York, US	15	1	Admitted to hospital	June 25th 2020
Hameed et al. (18)	Children with PIMS-TS	London, UK	35	1	Admitted to hospital	June 25th 2020
Feldstein et al. (19)	Children with MIS-C	US	186^a^	53	Admitted to hospital	June 29th 2020
Dufort et al. (20)	Children with MIS-C	New York, US	99^b^	106	Admitted to hospital	June 29th 2020

*Overview of articles describing cases of PIMS-TS, SARS-CoV-2 related Kawasaki-like disease, or MIS-C [per 29-06-2020]; only articles with 5 or more cases included*.

*Search strategy: using MIS-C, PIMS-TS and Kawasaki Disease as search terms in Medline search, age range: 0–<21 years, from April 1st to June 29th 2020; only English articles were included; only peer reviewed and published articles were included, pre-print manuscripts were excluded*.

*MISC, Multisystem Inflammatory Syndrome in Children; PICU, Pediatric Intensive Care Unit; PIMS-TS, Pediatric Inflammatory Multisystem Syndrome-temporally associated with SARS-CoV-2*.

a *Feldstein et al. (19) excluded cases in the cohort described by Dufort et al. (20) (n = 27), but included cases reported by Chiotos et al. (7) (n = 6) and Waltuch et al. (21) (n = 4). Feldstein et al. (19): 186 cases meeting the case definition included out of 234 reported cases*.

b *Dufort et al. (20): details of 99 confirmed or suspected cases via Public Health reporting registry included, out of 191 potential reported cases*.

**Table 2:** Presenting signs and symptoms, treatment, and outcomes in children with PIMS-TS.

	**Whittaker et al. (11)**	**Feldstein et al. (19)**	**Dufort et al. (20)**
Age (median, IQR, years)	9 (5.7–14)	8.3 (3.3–12.5)	31 (31%) <5 years, 42 (42%) 6–12 years
Gender, female	33 (57%)	71 (38%)	46 (46%)
Comorbidities	7 (12%)	51 (27%)^e^	36 (36%)
Overweight, obesity^a^	–	45/153 (29%)	29 (29%)
Asthma	3 (5%)	33 (18%)^f^	12 (12%)^f^
Underlying cardiac disease	0 (0%)	5 (3%)	–
*Clinical signs and symptoms*			
Fever	58 (100%)	186 (100%)	99 (100%)
Duration of fever/symptoms (median, IQR, in days)	3–9 (range)	6 (5–8)	4 (3–6)
Shock^b^	29 (50%)	89 (48%)	10 (10%)
Gastro-intestinal symptoms	–	–^g^	79 (80%)
Abdominal pain	31 (53%)	–	60 (61%)
Diarrhea	30 (52%)	–	49 (49%)
Vomiting, nausea	26 (45%)	–	57 (58%)
Kawasaki features			
Kawasaki, complete^c^	7 (12%)	74 (40%)	–^j^
Rash	30 (52%)	110 (59%)	59 (60%)
Conjunctival injection, conjunctivitis	26 (45%)	103 (55%)	55 (56%)
Mucous membrane changes	17 (29%)	78 (42%)	27 (27%)
(Cervical) lymphadenopathy, >1.5 cm diameter	9 (16%)	18 (10%)	6 (6%)
Swollen hand and feet	9 (16%)		9 (9%)
Changes to extremities	–	69 (37%)	
Respiratory symptoms	12 (21%)	–^g^	40 (40%)^k^
Sore throat	6 (10%)	–	16 (16%)
Rhinorrhoea, nasal congestion	–	–	13 (13%)
Chest pain	–	–	11 (11%)
Neurological symptoms		–^g^	30 (30%)
Confusion, altered mental state	5 (9%)	–	2 (2%)
Headaches	15 (26%)	–	29 (29%)
Arthralgia, arthritis	–	4 (2%)	4 (4%)
Myalgia, myositis	–	15 (8%)	17 (17%)
*Virology results*			
Nasopharyngeal SARS-CoV-2 RT-PCR	15 (26%)	73 (56%)	50/98 (51%)
Positive SARS-CoV-2 serology	40/46 (83%)	85 (46%)	76/77 (99%)
No positive SARS-CoV-2 result^d^	13 (22%)	55 (30%)	4 (4%)
*Immunomodulatory treatment*			
Nil immunomodulatory drugs	13 (22%)	–	–
Intravenous immunoglobulins	41 (71%)	144 (77%)	69 (70%)
Steroids	37 (64%)	91 (49%)	63 (64%)
Biologicals			
Anakinra (anti-IL1)	3 (5%)	24 (13%)	–
Infliximab (anti-TNF-α)	8 (14%)	0 (0%)	–
Toculizumab (anti-IL6)	0 (0%)	14 (8%)	–
*Outcomes*			
Critical care admission	29 (50%)	148 (80%)	79 (80%)
Inotropic/vasopressor support	27 (47%)	89 (48%)	61 (62%)
Mechanical invasive ventilation	25 (43%)	37 (20%)	10 (10%)
Extracorporeal membrane oxygenation	3 (5%)	8 (4%)	4 (4%)
Coronary artery aneurysm (*z*-score >2)	8 (14%)	15 (8%)^h^	9 (9%)
Death^i^	1 (2%)	4 (2%)	2 (2%)

*This is a summary table of clinical signs and symptoms, treatment, and outcomes from the three largest cohorts published up until June 29th 2020. Appendix 1 in Supplementary Materials includes these data from all published cohorts with 10 or more cases (as per June 29th 2020)*.

*IL1, Interleukin 1; IL6, interleukin 6; IQR, Interquartile range; IVIG, intravenous immunoglobulins; RT-PCR, Reverse Transcriptase Polymerase Chain Reaction; SARS-CoV-2, severe acute respiratory syndrome coronavirus 2; TNF, Tumor Necrosis Factor*.

a *Feldstein et al. (19) report both clinically diagnosed obesity (n = 12/153, 8%) and BMI based obesity (n = 45/153, 29%)*.

b *Whittaker et al. (11): shock defined as needing inotrope support or fluid resuscitation >20 mL/kg; Feldstein et al. (19): as requiring vasopressors*.

c *Complete or classic Kawasaki Disease as defined by fever for ≥5 days plus four or more clinical criteria, including bilateral bulbar non-exudative conjunctivitis, changes of the lips or oral cavity, non-suppurative laterocervical lymphadenopathy, polymorphic rash, erythema of the palms and soles, firm induration of the hands or feet, or both, as per the American Heart Association Criteria (22). Whittaker et al. (11): a total of 13 children (22%) met the criteria for Kawasaki Disease when coronary artery aneurysms were included. Feldstein et al. (19): 74 patients (40%) had fever for at least 5 days and four or five Kawasaki disease–like features or two or three Kawasaki disease–like features plus additional laboratory or echocardiographic findings*.

d *Papers also report on epidemiological links with a close contact of suspected or confirmed SARS-CoV-2 contact for those children with negative SARS-CoV-2 diagnostic results*.

e *Feldstein et al. (19): this is number of children with one or more comorbidities, excluding obesity; other papers included obesity as comorbidity*.

f *Feldstein et al. (19): number of children with asthma included all children with respiratory comorbidity; Dufort et al. (20): additional 2 children had other respiratory comorbidities*.

g *Feldstein et al. (19): showed gastro-intestinal involvement in 170 (91%) children, respiratory insufficiency or failure in 109 (59%) children, and neurological complications (encephalitis, aseptic meningitis, demyelinating disorder, seizures, coma or unresponsive within 24 h of admission) in 10 (6%) children; 132 (71%) children had four or more organ systems involved, 36 (19%) children had three organ systems involved, 18 (10%) children had two organ systems involved*.

h *Feldstein et al. (19): z-score >2.5*.

i *Not all children had been discharged from hospital at the end of the study periods*.

j *Dufort et al. (20): a total of 36 patients (36%) received a diagnosis of Kawasaki Disease or atypical (or incomplete) Kawasaki Disease*.

k *Dufort et al. (20): including cough n = 31 (31%), shortness of breath n = 19 (19%), wheezing n = 1 (1%)*.

This statement has been written on behalf of both the Pediatric Section of the European Society for Emergency Medicine and the European Academy of Pediatrics to provide an update for health care professionals in primary or secondary care undertaking assessments of acutely unwell children. This statement aims to

- provide information about the Pediatric Inflammatory Multisystem Syndrome temporally associated with SARS-CoV-2 (PIMS-TS);
- give initial guidance on the clinical assessment and management of children suspected of this new condition for health care professionals dealing with acutely unwell children;
- point out useful resources on the recognition and management of these children.

This statement does not address the management of children with PIMS-TS admitted under specialist care in the fields of infectious diseases, cardiology, or critical care.

Historically, a link between coronaviruses and Kawasaki syndrome was first proposed in 2005 (23). However, a causal relation has thus far not been proven, and the cause of Kawasaki Disease is currently unknown (24, 25). Awareness of the new hyperinflammatory condition affecting children was raised by clinicians in the UK, leading to an alert by the UK National Health Service, on April 25th 2020. Since then many other countries have issued similar alerts with guidance on management of these children. The first case report of a child with Kawasaki-like disease was reported in the international literature on April 22nd (26). The first case series with cases from the UK was reported on May 7th (5), followed by similar case series from Italy on May 13th **[**9]. Cases have now been reported throughout Europe and North America, with the European Center for Disease Control providing an up to date overview (27). The European Society for Medicine and the European Academy of Pediatrics hosted informative webinars (links in Table 3).

**Table 3:** Available resources, guidelines and ongoing studies.

**Resources and guidelines**
European Center for Disease Prevention and Control (ECDC)
https://www.ecdc.europa.eu/en/publications-data/paediatric-inflammatory-multisystem-syndrome-and-SARS-CoV-2-rapid-risk-assessment
British Pediatric Allergy, Immunity and infection Group (UK)
https://www.bpaiig.org/news-bpaiig-position-statement-SARS-CoV-2-treatment-guidance-version-12
Royal College of Pediatrics and Child Health (UK)
https://www.rcpch.ac.uk/resources/guidance-paediatric-multisystem-inflammatory-syndrome-temporally-associated-covid-19
Don't forget the bubbles
https://dontforgetthebubbles.com/pims-ts/
World Health organization (WHO)
https://www.who.int/news-room/commentaries/detail/multisystem-inflammatory-syndrome-in-children-and-adolescents-with-covid-19
American Academy of Pediatrics (US)
https://www.aappublications.org/news/2020/05/14/covid19inflammatory051420?cct=2287
Center of Disease Control and prevention (US)
https://www.cdc.gov/coronavirus/2019-ncov/hcp/pediatric-hcp.html#anchor_1589580133375
https://emergency.cdc.gov/han/2020/han00432.asp
PENTA network
https://penta-id.org/covid-19-outbreak/
European Society of Medicine
https://eusem.org/news/corona-virus
European Academy of Pediatrics
https://www.eapaediatrics.eu/covid-19-resource-centre/
**PIMS-TS studies and registries**
*Clinical data collection:*
ISARIC study, International Severe Acute Respiratory and emerging Infection Consortium: https://isaric.tghn.org
British Pediatric Surveillance Unit (UK)
https://www.rcpch.ac.uk/work-we-do/bpsu/study-multisystem-inflammatory-syndrome-kawasaki-disease-toxic-shock-syndrome
World Health organization (WHO)
https://www.who.int/news-room/commentaries/detail/multisystem-inflammatory-syndrome-in-children-and-adolescents-with-covid-19
*Diagnostics and -omics studies:*
DIAMONDS study, Diagnosis and Management of Febrile Illness using RNA Personalized Molecular Signature Diagnosis (Europe):
https://www.diamonds2020.eu
*Treatment studies:*
Registry: Best Available Treatment Study for the Pediatric Inflammatory Multisystem Syndrome temporally associated with SARS-CoV-2 (BATS-study):
https://bestavailabletreatmentstudy.co.uk
Clinical trial: RECOVERY trial, Randomized Evaluation of COVID-19 Therapy:
https://www.recoverytrial.net

The initial guidance from the Royal College of Pediatrics and Child Health in the United Kingdom provided a case definition and called this emerging disease entity the Pediatric Inflammatory Multisystem Syndrome—temporally associated with SARS-CoV-2 (PIMS-TS) (28). In the United States this disorder is referred to as the multisystem inflammatory syndrome in children (MIS-C) (29). By now, three case definitions are in use (Table 4). We recommend adhering to the case definition by the World Health Organization, as at present this definition appears to capture the spectrum of presenting signs and symptoms best (30). All current bodies proposing case definitions acknowledge the rapidly developing evidence and the need for continued evolvement of the case definitions.

**Table 4:** Case definitions of PIMS-TS (or MIS-C).

	**Population**	**Clinical signs and symptoms**	**Evidence of multiorgan involvement**	**Markers of inflammation**	**Evidence of other infections**	**Evidence of SARS-CoV-2 infection**	**Additional comments**
World Health Organization (30)	Children and adolescents 0–19 years of age	Fever > 3 days And two of the following:^∧^ Rash or bilateral non-purulent conjunctivitis or muco-cutaneous inflammation signs (oral, hands, or feet). Hypotension or shock. Acute gastrointestinal problems (diarrhea, vomiting, or abdominal pain)	Features of myocardial dysfunction, pericarditis, valvulitis, or coronary abnormalities, including echocardiogram findings or elevated Troponin/NT-proBNP Evidence of coagulopathy (by PT, APTT, elevated d-Dimers)	Elevated markers of inflammation such as erythrocyte sedimentation rate, C-reactive protein, or procalcitonin	No other obvious microbial cause of inflammation, including bacterial sepsis, staphylococcal, or streptococcal shock syndromes	Evidence of COVID-19 (RT-PCR, antigen test, or serology positive), or likely contact with patients with COVID-19	
Center of Disease Control and Prevention (CDC) (US) (29)	An individual under 21 years	Presenting with fever The CDC note the fever should be at least 38 degrees Celsius for at least 24 h or a subjective fever lasting 24 h	Evidence of clinically severe illness requiring hospitalization with multisystem (>2) organ involvement (cardiac, renal, respiratory, hematologic, gastrointestinal, dermatologic, or neurological)	Evidence of inflammation could include but is not limited to an elevated C-reactive protein, erythrocyte sedimentation rate, fibrinogen, procalcitonin, d-dimer, ferritin, lactic acid dehydrogenase, or interleukin 6, elevated neutrophils, reduced lymphocytes, and low albumin	No alternative plausible diagnoses	Positive for current or recent SARS-CoV-2 infection by reverse-transcriptase polymerase chain reaction, serology or antigen test; or COVID-19 exposure within the 4 weeks prior to the onset of symptoms	Some individuals may fulfill full or partial criteria for Kawasaki disease but should be reported if they meet the case definition for MIS-C Consider MIS-C in any pediatric death with evidence of SARS-CoV-2 infection
Royal College of Pediatrics and Child Health (UK) (28)	Any child	Persistent fever	Evidence of single or multi-organ dysfunction (shock, cardiac, respiratory, renal, gastrointestinal or neurological disorder) with other additional clinical, laboratory or imagining, and ECG features	Neutrophilia, elevated CRP, and lymphopaenia	Exclusion of any other microbial cause, including bacterial sepsis, staphylococcal or streptococcal shock syndromes, infections associated with myocarditis such as enterovirus	SARS-CoV-2 PCR testing positive or negative	Children fulfilling full or partial criteria for Kawasaki disease may be included

∧ *Two of the following of clinical signs and symptoms, or evidence of multiorgan involvement*.

The three case definitions have in common that they describe a population of children at risk with (1) persistent fever, (2) clinical signs and biochemical profiles reflecting ongoing inflammation, (3) the potential of multiorgan involvement (and most importantly the risk of cardiac involvement), (4) the absence of other reasonable explanations of the acute illness, and (5) evidence of a preceding SARS-CoV-2 infection or exposure to a suspected or confirmed case. Notably, children of all ages appear affected, but it has been more commonly reported in the adolescent age group, which is distinctly different from the children with classic Kawasaki Disease and children with toxic shock syndrome (5, 11). Also, boys and girls appear affected similarly (Table 2, Appendix 1 in Supplementary Materials), whereas children of black and minority ethnicity backgrounds appear affected more often (4, 9, 11).

It is important to stress that the incidence of PIMS-TS is rare with an estimated incidence at 2 in 100,000 persons <21 years of age (20). Also, although some deaths in children with PIMS-TS have been reported, as well as children requiring extra-corporeal membrane oxygenation (27), many affected children don't require critical care and have a full, and rapid clinical recovery (Table 2, Appendix 1 in Supplementary Materials).

There appears to be a wide spectrum of signs and symptoms with varying degree of severity (Table 2, Appendix 1 in Supplementary Materials). All children present with persistent fever. Typically, a substantial proportion of children present with abdominal symptoms, with some having symptoms of such severity that they had US and CT imaging, and some undergoing surgical procedures (10, 11, 31). The PIMS-TS spectrum includes disease entities that need urgent recognition and treatment, such as a toxic shock like presentation with hypovolaemia and shock, as well as Kawasaki-like disease with involvement of the coronary arteries (4–6). Children with Kawasaki-like disease will have all or some of the typical features, such as a rash and skin changes, conjunctival injection, mucous membrane changes, unilateral lymphadenopathy, and swollen hands and feet (6, 11). A majority of the reported children with PIMS-TS had evidence of multiorgan involvement (19). A number of these children will subsequently need critical care with (multiorgan) supportive care, whereas others can be managed safely on normal pediatric wards. Almost exclusively, these children appear ill and present in a manner that warrants enough concern to admit them to hospital and to perform additional diagnostic tests. By now, it appears that the biochemical profile of children with PIMS-TS is distinct from that of children with classic Kawasaki Disease and Kawasaki Disease with shock, with children with PIMS-TS having noticeably higher markers of inflammation (e.g., CRP, ferritin), marked lymfopaenia, greater elevation of troponin, and higher levels of fibrinogen; it is less clear how the biochemical profiles differ from children with toxic shock syndrome (11). Lastly, it is important to note, however, that the cases reported in the literature are likely to represent the more severe spectrum of disease.

Some of these children test positive for SARS-CoV-2 on PCR, and most have evidence of a previous infection with positive SARS-CoV-2 serology (Table 2, Appendix 1 in Supplementary Materials); however, some cases do not have a history of exposure to SARS-CoV-2 and do not have clear evidence of a (previous) SARS-CoV-2 infection (11). It remains important to perform (serial) tests to confirm the presence of active or previous infection with SARS-CoV-2, and to further our understanding of the relationship between and the underlying mechanisms of infection with SARS-CoV-2 and PIMS-TS (32).

After a reduction in the number of presentations to emergency departments during the lockdown periods across Europe (33), the raised awareness of PIMS-TS in the media might lead to an increase of presentations of children with fever and other infectious symptoms to acute care facilities. Public health bodies should communicate clearly with the general public about the warning signs of PIMS-TS and about when to seek care as to prevent delayed presentations (34, 35). Moreover, an increase in incidence of PIMS-TS has a 4–6 weeks delay after the onset of a COVID-19 outbreak (19, 20, 36).

As with any emerging disease, it might not immediately be straightforward deciding who is at risk of PIMS-TS, and in whom to perform additional investigations. This could adversely lead to false positive test results creating clinical uncertainty, erroneous clinical decision making, and heightened parental anxiety. Similarly, there is a risk that children will be classified as having sepsis, exacerbated by the risk of delayed presentations during a pandemic (34), and that they will be managed as such in their local hospital, without appropriate investigations and escalation of care. There is also a risk that many children with other, more common, childhood infections will be classified as suspected PIMS-TS and will undergo unnecessary diagnostic tests and treatment. For children with abdominal symptoms, an early diagnosis of PIMS-TS may inadvertently lead to a delay in surgical review, and concerns of surgical abdominal pathology could inversely lead to a delay in recognition of a diagnosis of PIMS-TS. Involvement of senior clinical decision makers and adherence to up-to-date case definitions and guidance are advisable.

Altogether, the emergence of PIMS-TS dictates careful clinical decision making when dealing with febrile children presenting to acute care facilities across Europe:

1. The need for additional investigations should be based on the initial clinical assessment of a febrile child by a clinician experienced in pediatric care.
2. The management of febrile children who appear clinically well, with a clear focus of infection, and who would previously have been deemed well enough for discharge from our care without any interventions should generally not change; this is likely to reflect the management of the vast majority of febrile children presenting to acute care facilities.
3. Ensure that the presence or absence of all Kawasaki-like features are documented for all febrile children; ensure that a blood pressure and a full set of vital parameters are recorded.
4. Clinicians should be aware of and follow published guidance on PIMS-TS by (inter)national societies (Table 3).
5. Perform additional investigations at an early stage in unwell appearing febrile children with sufficient heightened clinical concern as based on the case definition, or with clinical signs of inflammation and/or shock.
6. Additional laboratory investigations should include markers of myocardial involvement (such as troponin, pro-BNP), hypercoagulation (including APTT, PT, fibrinogen, D-Dimer), markers of inflammation (such as C-reactive protein, procalcitonin, ferritin, IL-6, erythrocyte sedimentation rate), creatine kinase, lactate dehydrogenase, full blood count, renal profile, vitamin D level, and liver function tests, including triglycerides; include amylase in the presence of abdominal symptoms (Table 5).
7. Request appropriate microbiology, including a blood culture, and virology tests to rule out any other infectious cause of the illness (Table 5). We also suggest PCR of oropharyngeal swab for SARS-CoV-2 in first instance. Save an EDTA and serum sample for PCR and serology studies prior to giving intravenous immunoglobulins.
8. An ECG and echocardiogram should be part of the diagnostic work-up of all children with suspected PIMS-TS.
9. We recommend early discussion with a specialist pediatric infectious diseases team if there is clinical and biochemical evidence of inflammation and suspicion of PIMS-TS. We also recommend early discussion with a pediatric critical care team in children in need for single or multiple organ support. Any evidence of myocardial involvement (for example, raised troponin or pro-BNP, or concerning ECG or echocardiogram) warrants early escalation to a center with pediatric cardiology expertise.

**Table 5:** List of investigations in PIMS-TS.

Blood tests	Blood gas with lactate Full blood count and film Renal profile Liver function tests, LDH, Triglycerides, CK Amylase in presence of abdominal symptoms C-reactive protein, procalcitonin, ferritin, ESR Coagulation screen with fibrinogen and D-Dimer Troponin, pro-BNP Vitamin D
Imaging	Chest X ray ECG, echocardiogram Abdominal US with abdominal symptoms
Microbiology and virology	Blood culture Urine culture Stools culture Throat culture Nasopharyngeal aspirate or throat swab for PCR for respiratory viruses *Mycoplasma pneumoniae* titres, ASOT PCR for bacterial pathogens (e.g., meningococcal, pneumococcal, streptococcal, and staphylococcal PCR) on blood PCR for EBV, CMV, adenovirus, parvovirus, enterovirus, and parechovirus on blood HIV Save samples for PCR and serology studies (prior to IVIG)
SARS-CoV-2 investigations	SARS-CoV-2 Respiratory PCR; *Consider PCR on stools and blood* SARS-CoV-2 serology

*Proposed list of initial investigations in children with suspected PIMS-TS needing hospital admission. This list is not exhaustive and additional investigations should be tailored to each individual patient*.

*Anti-Streptolysin O Titer; CK, Creatine Kinase; EBV, Epstein-Barr Virus; ECG, electrocardiogram; ESR, Erythrocyte Sedimentation Rate; HIV, Human Immunodeficiency Virus; LDH, lactate dehydrogenase; PCR, Polymerase Chain Reaction; pro-BNP, pro-Brain Natriuretic Peptide; SARS-CoV-2, severe acute respiratory syndrome coronavirus 2; US, Ultrasound*.

This statement does not cover the specific treatment of individual children with PIMS-TS; all these children should be discussed with a pediatric expertise center. At present, there is no high-level evidence to support any best care recommendations about the treatment of these children outside providing optimal supportive care. For children with Kawasaki-like disease, the mainstay of treatment consists of intravenous immunoglobulins, steroids and possibly additional biologicals, such as anti-TNF, anti-IL1, or anti-IL6, and aspirin. For children presenting with a toxic shock like picture, treatment should focus on early cardiovascular support, treatment and reversal of shock, and intravenous immunoglobulins. Fluid boluses should be titrated carefully in view of the risk of myocardial impairment; inotropes and vasopressors should be started early as per established advanced life support guidelines (37). Another clinical picture describing patients with both Kawasaki-like disease plus shock will need aggressive treatment for both. Standards of antimicrobial stewardship should be upheld, but it will be difficult to withhold broad spectrum antibiotics as part of initial emergency care in most instances, and secondary bacterial infections have been reported. Additionally, many of these children will have evidence of hypercoagulation, and prophylactic or therapeutic anti-coagulation should be considered early in all children with PIMS-TS. It is crucial to highlight that treatment is time critical and should not be delayed. Immunomodulatory treatments should ideally be given in the context of a controlled trial, but it might prove difficult to set up and enroll children in randomized controlled trials for the treatment of PIMS-TS in the near future. The UK RECOVERY trial (Randomized Evaluation of COVID-19 Therapy) is currently the largest randomized controlled trial including children (38). Other international registries are aiming to capture clinical data (e.g. ISARIC study) and the use of different treatments (e.g. BATS study) around the world (Table 3).

A concern of the treatment with immunoglobulins, or convalescent plasma, is the unexplored potential to trigger an antibody mediated response, similar to the one that potentially underlies this hyperinflammatory syndrome.

Recommendations for the intermediate and long-term follow-up of children with PIMS-TS after hospitals discharge should be guided by the tertiary specialty teams. Special consideration should be given to the need of performing post-discharge echocardiograms to exclude late cardiac complications (15).

## Recommendations by the Pediatric Section of the European Society of Emergency Medicine and the European Academy of Pediatrics

1. Parents should seek medical attention if their child is unwell, develops any warning signs of fever as described by the traffic light system by the National Institute of Health and Care Excellence, or has a persistent fever for more than 5 days, to avoid delayed presentation (39).
2. Health care professionals in primary and secondary care looking after acutely unwell children are urged to take notice of the emerging PIMS-TS (Pediatric Inflammatory Multisystem Syndrome—temporally associated with SARS-CoV-2) and to be vigilant, as serious sequelae might occur if not recognized and treated in a timely manner.
3. We recommend performing additional investigations as per published guidelines (Table 5) and to refer patients with severe manifestations to specialist centers.
4. Children with PIMS-TS appear to respond well to treatment if recognized promptly with no delay in treatment, and most of the children will have a full and quick recovery. Thus, we urge all primary and secondary care health care professionals to include the diagnosis of PIMS-TS in the differential diagnosis of children with persistent fever for more than 5 days.
5. We recommend that clinicians document and share the data of their patients using research registries (Table 3) to study the full clinical spectrum of presenting signs and symptoms, ascertaining biochemical profiles of these children, validate predictors of disease progression, and to monitor treatment response (40).

## Public and Patient Involvement

This statement was derived without input from patients or representatives of the general public.

## Author Contributions

All authors listed have made a substantial, direct and intellectual contribution to the work, and approved it for publication.

## Conflict of Interest

The authors declare that the research was conducted in the absence of any commercial or financial relationships that could be construed as a potential conflict of interest.

## Abbreviations

Anti-IL1: anti-Interleukin 1
Anti-IL6: anti-Interleukin 6
Anti-TNF: anti-Tumor Necrosis Factor
APTT: Activated Partial Thromboplastin Time
ECG: Electrocardiogram
ESR: Erythrocyte Sedimentation Rate
PCR: Polymerase Chain Reaction
PT: Prothrombin Time
IL-6: Interleukin-6
PIMS-TS: Pediatric Inflammatory Multisystem Syndrome-temporally associated with SARS-CoV-2
pro-BNP: pro-Brain Natriuretic Peptide
SARS-CoV-2: severe acute respiratory syndrome coronavirus 2.
