# Supplementary material for: Pediatric Inflammatory Multisystem Syndrome: Statement by the Pediatric Section of the European Society for Emergency Medicine and European Academy of Pediatrics
Source: Front Pediatr. 2020 Aug 28;8:490. doi: 10.3389/fped.2020.00490 (PMC7485110; doi:10.3389/fped.2020.00490)
Supplement: Supplementary file 1 [file Data_Sheet_1.docx]

## Appendix 1. Overview of studies reporting children with PIMS-TS (with five or more cases).

|  | Population | Location | Number of children in study | Number of  settings | Eligibility | Date of publication |
| --- | --- | --- | --- | --- | --- | --- |
| Verdoni *et al*.^1^ | Children with Kawasaki-like disease | Bergamo, Italy | 10 | 1 | admitted to the general paediatric unit | May 13^th^ 2020 |
| Riphagen *et al.*^16^ | Children with hyperinflammatory shock | London, UK | 8 | 1 | Admitted to PICU | May 6^th^ 2020 |
| Zahra *et al.*^2^ | Children with acute heart failure in MIS-C | France, Switzerland | 35 | 14 | admitted to PICU | May 17^th^ 2020 |
| Chiotos *et al.*^3^ | Children with MIS-C | Philadelphia, US | 6 | 1 | admitted to PICU | May 28^th^ 2020 |
| Grimaud *et al.*^3^ | Acute myocarditis and multisystem inflammatory emerging disease in critically ill children | Paris, France | 20 | 4 | admitted to PICU | June 1^st^ 2020 |
| Toubiana *et al.*^4^ | Children with Kawasaki-like multisystem inflammatory syndrome | Paris, France | 21 | 1 | admitted to general paediatric ward | June 3^rd^ 2020 |
| Miller *et al*.^5^ | Children with MIS-C | New York, US | 44 | 1 | admitted to hospital | June 4^th^ 2020 |
| Whittaker *et al.*^6^ | Children with PIMS-TS | United Kingdom | 58 | 8 | admitted to hospital | June 8^th^ 2020 |
| Cheung *et al.* ^7^ | Children with MIS-C | New York, US | 17 | 1 | admitted to hospital | June 8^th^ 2020 |
| Capone *et al.*^8^ | Children with MIS-C | New York, US | 33 | 1 | admitted to hospital | June 10^th^ 2020 |
| Pouletty *et al.* ^9^ | Children with PIMS-TS mimicking Kawasaki disease (Kawa-COVID-19) | Paris, France | 16 | 7 | Admitted to hospital | June 11^th^ 2020 |
| Ramcharan *et al.*^10^ | Children with PIMS-TS | Birmingham, UK | 15 | 1 | admitted to hospital | June 12^th^ 2020 |
| Kaushik *et al.*^11^ | Children with MIS-C | New York, US | 33 | 3 | admitted to PICU | June 14^th^ 2020 |
| Riollano-Cruz *et al.*^12^ | Children with MIS-C | New York, US | 15 | 1 | admitted to hospital | June 25^th^ 2020 |
| Hameed et al.^13^ | Children with PIMS-TS | London, UK | 35 | 1 | admitted to hospital | June 25^th^ 2020 |
| Feldstein *et al.*^14^ | Children with MIS-C | US | 186^a^ | 53 | admitted to hospital | June 29^th^ 2020 |
| Dufort *et al.* ^15^ | Children with MIS-C | New York, US | 99^b^ | 106 | admitted to hospital | June 29^th^ 2020 |

*Legend:*

Overview of articles describing cases of PIMS-TS, SARS-CoV-2 related Kawasaki-like disease, or MIS-C [per 29-06-2020]; only articles with 5 or more cases included.

Search strategy: using MIS-C, PIMS-TS and Kawasaki Disease as search terms in Medline search, age range: 0 - <21 years, from April 1^st^ – June 29^th^ 2020; only English articles included; only peer reviewed and published articles included, pre-print manuscripts were excluded.

^a^ Feldstein *et al.* excluded cases in the cohort described by Dufort *et al.* (n=27), but included cases reported by Chiotos *et al.*^17^ (n=6) and Waltuch *et al.*^18^ (n=4). Feldstein *et al.*: 186 cases meeting the case definition included out of 234 reported cases.

^b^ Dufort *et al.:* details of 99 confirmed or suspected cases via Public Health reporting registry included, out of 191 potential reported cases

## Appendix 1. Summary evidence tables

|  | Verdoni *et al*.^1^ | Zahra *et al.*^2^ | Grimaud *et al.*^3^ | Toubiana *et al.*^4^ | Miller *et al*.^5^ | Whittaker *et al.*^6^ | Cheung *et al.* ^7^ | Capone *et al.*^8^ |
| --- | --- | --- | --- | --- | --- | --- | --- | --- |
| Age (median, IQR, years) | 7.5, range 2.9-16.0 | 10 (-) | 10, range 2.9-15 | 7.9, range 3.7-16.6 | 7.3, range 7m-20y | 9 (5.7 – 14) | 8, range 1.1-16 | 8.6 (5.5-12.6) |
| Gender, female | 3 (30%) | 17 (49%) | 10 (50%) | 12 (57%) | 24 (55%) | 33 (57%) | 9 (53%) | 13 (39%) |
| Comorbidities | - | 10 (28%) | 0 (0%) | 0 (0%) | - | 7 (12%) | - | 7 (21%) |
| Overweight, obesity^a^ | - | 6 (17%) | - | 5 (24%) | 16 (39%) | - | - | 15 (45%) |
| Asthma^b^ | - | 3 (9%) | - | - | - | 3 (5%) | 3 (18%) | 5 (15%) |
| Underlying cardiac disease | - | 0 (0%) | - | - | - | 0 (0%) | - | - |
| *Clinical signs and symptoms* |  |  |  |  |  |  |  |  |
| Fever | 10 (100%) | 35 (100%) | 20 (100%) | 21 (100%) | 44 (100%) | 58 (100%) | 17 (100%) | 33 (100%) |
| Duration of fever or symptoms (median, IQR, in days) | 4 – 8 (range) | - | 6 (1-10) | - | - | 3 - 19 (range) | 5 (1-12, range) | 4 (3 – 5) |
| Shock^c^ | 5 (50%) | 28 (80%) | 20 (100%) | 12 (57%) | 22 (50%) | 29 (50%) | 13 (76%) | 25 (76%) |
| Gastro-intestinal symptoms | - | 29 (83%) | - | 21 (100%) | 37 (84%)^j^ | - | 15 (88%) | 32 (97%) |
| Abdominal pain | - | - | 20 (100%) | - | 33 (75%) | 31 (53%) | - | - |
| Diarrhoea | 6 (60%) | - | - | - | 18 (40%) | 30 (52%) | - | - |
| Vomiting, nausea | - | - | - | - | 25 (57%) | 26 (45%) | - | - |
| Kawasaki features |  |  |  |  |  |  |  |  |
| Kawasaki, Complete^d^ | 5 (50%) | 0 (0%) | 0 (0%) | 11 (52%) | - | 7 (12%) | 8 (47%) | 21 (64%) |
| Rash | - | 20 (57%) | 10 (50%) | 16 (76%) | 31 (71%) | 30 (52%) | 12 (71%) | - |
| Conjunctival injection, conjunctivitis | - | - | 6 (30%) | 17 (81%) | 23 (52%) | 26 (45%) | 11 (65%) | - |
| Mucous membrane changes | - | - | 5 (25%) | 16 (76%) | 23 (52%) | 17 (29%) | 9 (53%)^r^ | - |
| (Cervical) lymphadenopathy | - | 21 (60%) | 2 (10%) | 12 (57%) | - | 9 (16%) | - | - |
| Swollen hand and feet | - | - | - | - | - | 9 (16%) | - | - |
| Changes to extremities | - | - | - | 10 (48%)^h^ | - | - | 3 (18%)^r^ | - |
| Upper Respiratory symptoms | - | - | - | - | - | 12 (21%) | 7 (41%) | 17 (52%)^q^ |
| Sore throat | - | - | - | - | - | 6 (10%) | - | - |
| Rhinorrhoea, nasal congestion | - | 15 (43%) | - | - | - | - | - | - |
| Lower respiratory symptoms |  | 23 (65%) | - | - | - | - | - | - |
| Cough | - | - | - | - | - | - | - | - |
| Shortness of breath | - | - | - | - | - | - | - | - |
| Wheezing | - | - | - | - | - | - | - | - |
| Hypoxia | - | - | - | - | 11 (25%) | - | 3 (18%) | - |
| Chest pain | - | 6 (17%) | - | - | - | - | - | - |
| Neurological symptoms | - | 11 (31%) | - | 6 (29%)^i^ | 13 (30%)^i^ | - | - | 19 (58%)^i^ |
| Confusion, altered mental state | - | - | -^i^ | - | - | 5 (9%) | - | - |
| Irritability | - | - | - | 12 (57%) | ^-^ |  | - | ^-^ |
| Headaches | - | - | - | - | - | 15 (26%) | - | - |
| Meningism | 4 (40%) | - | - | - | - | - | - | - |
| Asthenia | - | 35 (100%) | - | - | - | - | - | - |
| Arthralgia, arthritis |  |  |  |  |  |  |  |  |
| Myalgia, myositis | - | - | - | 2 (10%) | - | - | - | - |
| *Virology results* |  |  |  |  |  |  |  |  |
| Nasopharyngeal SARS-CoV-2 RT-PCR | 2 (20%) | 12 (34%)^f^ | 10 (50%)^f^ | 8 (38%) | 15/44 (34%) | 15 (26%) | 8 (47%) | 6 (18%) |
| Positive SARS-CoV-2 serology | 8 (80%) | 30 (86%) | 15/15 (100%) | 19 (90%) | 31/32 (97%) | 40/46 (83%) | 9 (53%) | 30 (91%) |
| No positive SARS-CoV-2 result^e^ | 2 (20%) | 4 (11%) | 1 (5%) | 2 (10%) | 5 (11%) | 13 (22%) | 0 (0%) | 3 (9%) |
| *Immunomodulatory treatment* |  |  |  |  |  |  |  |  |
| Nil immunomodulatory drugs | 0 (0%) | - | 0 (0%) | (0%) | - | 13 (22%) | 0 (0%) | 0 (0%) |
| Intravenous immunoglobulins | 10 (100%) | 25 (71%) | 20 (100%) | 21 (100%) | 36 (82%) | 41 (71%) | 13 (76%) | 33 (100%) |
| Steroids | 8 (80%) | 12 (34%) | 2 (10%) | 10 (48%) | 42 (96%) | 37 (64%) | 14 (82%) | 23 (70%) |
| Biologicals |  |  |  |  |  |  |  |  |
| Anakinra (anti-IL1) | - | 3 (8%) | 1 (5%) | - | 8 (18%) | 3 (5%) | 0 (0%) | 4 (12%) |
| Infliximab (anti-TNF-α) | - | 0 (0%) | 1 (5%) | - | 0 (0%) | 8 (14%) | 0 (0%) | 1 (3%) |
| Toculizumab (anti-IL6) | - | 0 (0%) | 0 (0%) | - | 0 (0%) | 0 (0%) | 1 (6%) | 3 (9%) |
| *Outcomes* |  |  |  |  |  |  |  |  |
| Critical care admission | - | 35 (100%)^g^ | 20 (100%) | 17 (81%) | - | 29 (50%) | 15 (88%) | 26 (79%) |
| Inotropic/vasopressor support | 2 (20%) | 28 (80%) | 19 (95%) | 15 (71%) | 22 (50%) | 27 (47%) | 10 (59%) | 25 (76%) |
| Mechanical invasive ventilation | - | 22 (62%) | 8 (40%) | 11 (52%) | 1 (2%) | 25 (43%) | 0 (0%) | 6 (18%) |
| Extracorporeal membrane oxygenation | - | 10 (28%) | 0 (0%) | - | - | 3 (5%) | - | - |
| Coronary artery aneurysm (z-score >2) | 2 (20%) | 6 (17%) | 0 (0%) | 5 (24%) | - | 8 (14%) | 1 (6%) | 8 (24%)^n^ |
| Death^p^ | - | 0 (0%) | 0 (0%) | 0 (0%) | 0 (0%) | 1 (2%) | 0 (0%) | 0 (0%) |

## Appendix 1. Summary evidence tables, continued

|  | Pouletty *et al.*^9^ | Ramcharan *et al.*^10^ | Kaushik *et al.*^11^ | Riollano-Cruz *et al.*^12^ | Hameed et al.^13^ | Feldstein *et al.* ^14^ | Dufort *et al.* ^15^ |
| --- | --- | --- | --- | --- | --- | --- | --- |
| Age (median, IQR, years) | 10 (4.7-12.5) | 8.8 (6.4-11.2) | 10 (6-13) | Range 3-20 years | 11 (IQR 8) | 8.3 (3.3-12.5) | Range up to 21 years  31 (31%) <5 years, 42 (42%) 6-12 years |
| Gender, female | 8 (50%) | 4 (27%) | 13 (39%) | 4 (27%) | 8 (23%) | 71 (38%) | 46 (46%) |
| Comorbidities | 6 (38%) | - | 16 (48%) | 4 (27%) |  | 51 (27%)^l^ | 36 (36%) |
| Overweight, obesity^a^ | 4 (25%) | - | 2 (6%) | - |  | 45/153 (29%) | 29 (29%) |
| Asthma^b^ | 2 (13%) | - | 5 (15%) | 4 (27%) |  | 33 (18%) | 12 (12%) |
| Underlying cardiac disease | - | - | 2 (6%) | - |  | 5 (3%) | - |
| *Clinical signs and symptoms* |  |  |  |  |  |  |  |
| Fever | 16 (100%) | 15 (100%) | 31 (93%) | 15 (100%) | 33 (94%) | 186 (100%) | 99 (100%) |
| Duration of fever or symptoms (median, IQR, in days) | 5 (4-7) | 5 | 4.5 (3-6) | - | - | 6 (5-8) | 4 (3-6) |
| Shock^c^ | 11 (69%) | - | 21 (63%) | 13 (87%) | 21 (60%) | 89 (48%) | 10 (10%) |
| Gastro-intestinal symptoms | 13 (81%) | 13 (87%) | - | 13 (87%) | 30 (86%) | -^m^ | 79 (80%) |
| Abdominal pain | - | - | 21 (63%) | - | - | - | 60 (61%) |
| Diarrhoea | - | - | 16 (48%) | - | - | - | 49 (49%) |
| Vomiting, nausea | - | - | 23 (69%) | - | - | - | 57 (58%) |
| Kawasaki features |  |  |  |  | - |  |  |
| Kawasaki, Complete^d^ | 10 (62%) | - | - | - | - | - | 40 (40%) |
| Rash | 13 (81%) | - | 14 (42%) | 7 (47%) | 13 (37%) | 110 (59%) | 59 (60%) |
| Conjunctival injection, conjunctivitis | 15 (94%) | - | 12 (36%) | 4 (27%) | 9 (26%) | 103 (55%) | 55 (56%) |
| Mucous membrane changes | 14 (87%)^r^ | - | 7 (21%) | - | - | 78 (42%) | 27 (27%) |
| (Cervical) lymphadenopathy | 6 (37%) | - | - | - | - | 18 (10%) | - |
| Swollen hand and feet | 11 (68%) | - | - | 4 (27%) | - |  | 9 (9%) |
| Changes to extremities |  | - | - | - | - | 69 (37%) |  |
| Upper Respiratory symptoms | - | - | 11 (33%) | - | - | - | 27 (27%) |
| Sore throat | - | - | - | - | - | - | 16 (16%) |
| Rhinorrhoea, nasal congestion | - | - | - | - | - | - | 13 (13%) |
| Lower respiratory symptoms | 2 (12%)^q^ |  |  | 3 (20%) | - | -^m^ | 40 (40%) |
| Cough | - |  |  | - | - | - | 31 (31%) |
| Shortness of breath | - |  |  | - | - | - | 19 (19%) |
| Wheezing | - |  |  | - | - | - | 1 (1%) |
| Hypoxia | - | - | - | - | - | - | - |
| Chest pain | - | - | - | 2 (13%) | - | - | 11 (11%) |
| Neurological symptoms | 9 (56%)^I,s^ | - | 4 (12)^i^ | - | - | -^m^ | 30 (30%) |
| Confusion, altered mental state | - | - | - | - | - | - | 2 (2%) |
| Irritability | - | ^-^ | ^-^ | - | - | - | - |
| Headaches | 6 (37%) | - | - | - | - | - | 29 (29%) |
| Meningism | - | - | - | - | - | - | - |
| Asthenia, lethargy | - | 4 (27%) | - | - | - | - | - |
| Arthralgia, arthritis | 1 (6%) | 4 (27%) |  | - | - | 4 (2%) | 4 (4%) |
| Myalgia, myositis | - | - | - | - | - | 15 (8%) | 17 (17%) |
| *Virology results* |  |  |  |  |  |  |  |
| Nasopharyngeal SARS-CoV-2 RT-PCR | 9 (56%)^f^ | 2 (13%) | 11 (33%) | 7 (47%) | 0 (0%) | 73 (56%) | 50 (51%) |
| Positive SARS-CoV-2 serology | 7/8 (87%) | 12/12 (100%) | 27 (81%) | 15 (100%) | 27/30 (90%) | 85 (46%) | 76/77 (99%) |
| No positive SARS-CoV-2 result^e^ | 2 (13%) | - | 0 (0%) | 0 (0%) | 8 (23%) | 55 (30%) | 4 (4%) |
| *Immunomodulatory treatment* |  |  | ^o^ | ^o^ |  |  |  |
| Nil immunomodulatory drugs |  |  | - |  | - | - | - |
| Intravenous immunoglobulins | 15 (93%) | 10 (67%) | 18 (54%) | 12 (80%) | - | 144 (77%) | 69 (70%) |
| Steroids | 4 (25%) | 5 (33%) | 17 (51%) | 3 (20%) | - | 91 (49%) | 63 (64%) |
| Biologicals |  |  |  |  | - |  |  |
| Anakinra (anti-IL1) | 1 (6%) | 0 (0%) | 4 (12%) | 2 (13%) | - | 24 (13%) | - |
| Infliximab (anti-TNF-α) | 0 (0%) | 0 (0%) | 0 (0%) | 0 (0%) | - | 0 (0%) | - |
| Toculizumab (anti-IL6) | 1 (6%) | 0 (0%) | 12 (36%) | 12 (80%) | - | 14 (8%) | - |
| *Outcomes* | ^0^ |  |  |  |  |  |  |
| Critical care admission | 7 (44%) | 10 (67%) | 33 (100%) | 14 (93%) | 24 (69%) | 148 (80%) | 79 (80%) |
| Inotropic/vasopressor support | 6 (38%) | 10 (67%) | 17 (51%) | 9 (60%) | 20 (57%) | 89 (48%) | 61 (62%) |
| Mechanical invasive ventilation | 2 (28%) | 4 (27%) | 5 (15%) | 3 (20%) | 7 (20%) | 37 (20%) | 10 (10%) |
| Extracorporeal membrane oxygenation | - | 0 (0%) | 1 (3%) | 1 (7%) | 2 (6%) | 8 (4%) | 4 (4%) |
| Coronary artery aneurysm (z-score >2) | 3 (19%) | 7 (47%)^k^ | 2 (6%)^n^ | - | 6 (17%) | 15 (9%)^n^ | 9 (9%) |
| Death^p^ | 0 (0%) | 0 (0%) | 1 (3%) | 1 (7%) | 1 (3%) | 4 (4%) | 2 (2%) |

*Legend:*

This is a summary table of the data on clinical signs and symptoms, treatment and outcomes as provided by the original publications; this table is not exhaustive and we refer to the individual papers for additional data. Only cohorts with 10 cases or more included in this evidence table.

Search strategy: using MIS-C, PIMS-TS and Kawasaki Disease as search terms in Medline search, age range: 0 - <21 years, from April 1^st^ – June 29^th^ 2020; only English articles included; only peer reviewed and published articles included, pre-print manuscripts were excluded.

Duplicate reporting of cases:

Feldstein *et al.* ^14^ excluded cases in the cohort described by Dufort *et al.* (n=27), but included cases reported by Chiotos *et al.*^17^ (n=6) and Waltuch *et al.*^18^ (n=4). Miller *et al*., Cheung *et al.*, Capone *et al.* all report cases from New York, US that might be duplicated in the study by Dufort *et al.*

Cases admitted to the PICU from the Necker Hospital for Sick Children in Paris, France, were described in three papers; Zahra *et al.* included cases between March 22^nd^ and April 30^th^; Toubiana et al. describes cases from April 27^th^ – May 11^th^ ; Grimaud *et al.* between April 15^th^ and April 27^th^. Pouletty *et al.* included patients in the Paris region but did not report publishing these cases elsewhere

The cohort described by Riphagen *et al.*^16^ (n=8, published May 7^th^ 2020) were also included in the cohort described by Whittaker *et al.*; It is not clear if and which patients in the paper by Hameed *et al.* and Ramcharan *et al.* were included in the paper by Whittaker *et al*.

IL1 Interleukin 1; IL6 interleukin 6; IQR Interquartile range; IVIG intravenous immunoglobulins; PICU Paediatric Intensive Care Unit; RT-PCR Reverse Transcriptase Polymerase Chain Reaction; SARS-CoV-2 severe acute respiratory syndrome coronavirus 2; TNF Tumor Necrosis Factor

^a^ Overweight: Zahra *et al.*: Body Mass Index >25 kg/m^2^,; Toubiana *et al.*: ≥97^th^ centile; Kaushik *et al*.: obesity defined as BMI>30 kg/m^2^, 4 (12%) children with overweight, 2 (6%) children were obese; Miller *et al.*: >85^th^ centile; Capone *et al.* 2 children BMI >=85^th^ centile, 13 children with BMI >= 95^th^ centile; Feldstein *et al.* report both clinically diagnosed obesity (n=12/153, 8%) and BMI based obesity (n=45/153, 29%).

^b^ Feldstein *et al.*: number of children with asthma included all children with respiratory comorbidity; Dufort *et al.*: additional 2 children had other respiratory comorbidities

^c^ Shock defined as: Verdoni *et al.*: hypotension and/or signs of hypoperfusion; Zahra *et al.*: cardiogenic shock with collapse; Grimaud *et al.*: tachycardia and one of the following signs: arterial systemic, hypotension, cold extremity, decrease peripheral pulse,, capillary refill time > 3 s, oliguria or arterial blood lactate, > 2 mmol/L, and shock was inclusion criterion; Toubiana *et al.*: needing fluid resuscitation and/or inotropic support; Whittaker *et al*: needing inotrope support or fluid resuscitation >20 mL/kg; Cheung *et al.:* not defined; Kaushik *et al.* as hypotension; Miller *et al*. and Capone *et al.* and Feldstein *et al.*: as requiring vasopressors; Riollano-Cruz *et al.*: tachycardia and hypotension; Pouletty *et al.*: haemodynamic failure defined as (ie, tachycardia and one of the following signs: arterial systemic hypotension, cold extremities, decreased peripheral pulse, capillary refill time >3 s, oliguria or blood lactate >2 mmol/L.

^d^ Complete or classic Kawasaki Disease as defined by fever for ≥5 days plus four or more clinical criteria, including bilateral bulbar non-exudative conjunctivitis, changes of the lips or oral cavity, non-suppurative laterocervical lymphadenopathy, polymorphic rash, erythema of the palms and soles, firm induration of the hands or feet, or both, as per the American Heart Association Criteria. ^19^ Whittaker *et al.*: a total of 13 children (22%) met the criteria for Kawasaki Disease when coronary artery aneurysms were included; Ramcharan *et al*: 8 (53%) had features of Kawasaki disease not fulfilling diagnostic criteria; Feldstein *et al.*: 74 patients (40%) had fever for at least 5 days and four or five Kawasaki disease–like features or two or three Kawasaki disease–like features plus additional laboratory or echocardiographic findings; Dufort *et al.*: A total of 36 patients (36%) received a diagnosis of Kawasaki Disease or atypical (or incomplete) Kawasaki Disease

^e^ papers also report on epidemiological links with a close contact of suspected or confirmed SARS-CoV-2 contact for those children with negative SARS-CoV-2 diagnostic results.

^f^ Zahra *et al.*, Pouletty *et al.*, Grimaud *et al.*: there were also two children with PCR on stools positive for SARS-CoV-2, and with negative respiratory specimen PCR

^g^ Zahra *et al*.: 29/35 children were admitted directly to the PICU

^h^ Toubiana *et al.*: Perineal or face desquamation, n=4 (19%)

^I^ Grimaud *et al.*: reported Glasgow Coma Scale median 15, IQR 4-15; Toubiana *et al*.: Other neurological features, n=6 (29%), defined as headaches, confusion, or meningeal irritation; Kaushik *et al.* defined as ‘neuro involvement’; Miller *et al.*: neurologic, defined as headache, vision changes, altered mental status, meningitic signs, cranial nerve palsy; Capone *et al.:* defined as Neurocognitive symptoms (headache, irritability, lethargy); Pouletty e*t al*.: neurological signs include headaches and aseptic meningitis (n=3)

^j^ Miller *et al.*: other abdominal symptoms include haematemesis, n=1 (2%), hematochezia/melena n=2 (5%), constipation n=5 (11%)

^k^ Ramcharan *et al.*: N=6 had ectatic dilated coronaries with increased z-scores, N=1 had aneurysm.

^l^ Feldstein *et al.*: this is number of children with one or more comorbidities, excluding obesity; other papers included obesity as comorbidity.

^m^ Feldstein *et al.*: showed gastro-intestinal involvement in 170 (91%) children, respiratory insufficiency or failure in 109 (59%) children, and neurological complications (encephalitis, aseptic meningitis, demyelinating disorder, seizures, coma or unresponsive within 24 hours of admission) in 10 (6%) children; 132 (71%) children had four or more organ systems involved, 36 (19%) children had three organ systems involved, 18 (10%) children had two organ systems involved.

^n^ Feldstein *et al.*: z-score >2.5; Kaushik *et al.:* 2 children with ectasia, 6 with prominent coronary arteries on echocardiogram (no z-scores given). Capone *et al.*: 5 children with z-score >= 2.5, 3 children z-score 2-2.49.

^o^ Kaushik *et al*.: 1 patient received convalescent plasma therapy; n=o hydrochloroquine; n=7 (21%) remdesivir; Riollano-Cruz *et al.*: one patient also received convalescent plasma therapy; n=2 patients received remdesivir. Pouletty *et al.:* 1 patient also received hydrochloroquine in view of suspected SLE.

^p^ not all children had been discharged from hospital at the end of the study periods

^q^ Capone *et al.*: Respiratory symptoms defined as cough, congestion, dyspnea, sore throat. Pouletty *et al.*: respiratory signs as cough or dyspnea

^r^ Cheung *et al.*: mucous membrane involvement described as lip redness and swelling; changes to extremities defined as skin desquamation; Pouletty *et al.*: mucous membrane changes defined as dry cracked lips.

^s^ Pouletty *et al.*: other atypical symptoms observed included Raynaud syndrome (n=2), anosmia (n=1), orchitis (n=2)
